# Risk and Protective Factors for Suicidal Ideation and Attempts Among Older Adults in California, 2021–2024 Pooled Cross-Sectional Study

**DOI:** 10.3390/healthcare14162485

**Published:** 2026-08-11

**Authors:** Kristen R. Choi, Gery Ryan, Sarah E. Choi, Luisa R. Blanco

**Affiliations:** 1 Joe C. Wen School of Nursing, University of California, Los Angeles (UCLA), Los Angeles, CA 90095, USA; schoi@sonnet.ucla.edu; 2 Department of Health Policy and Management, Fielding School of Public Health, University of California, Los Angeles (UCLA), Los Angeles, CA 90095, USA; 3 Department of Health Systems Science, Bernard J. Tyson School of Medicine, Kaiser Permanente Southern California, Pasadena, CA 90095, USA; gery.w.ryan@kp.org; 4 School of Public Policy, Pepperdine University, Malibu, CA 90095, USA; luisa.blancoraynal@pepperdine.edu

**Keywords:** suicide, older adults, race and ethnicity, survey, risk

## Abstract

Background/Objectives: Older adults have high rates of suicide, but there is little research on age-specific correlates of suicide. The purpose of this study was to examine risk and protective factors for suicidal ideation and attempts across adult and older adult age groups in a racially diverse sample. Methods: Using a cross-sectional analysis of California Health Interview Survey (CHIS) data from 2021 to 2024 (*N* = 92,397) linked with 2020 CDC/ATSDR Social Vulnerability Index data, the analytic sample was 64,263 California adults selected by address-based sampling and stratified by age: 45–64 years (31.5% of the overall pooled sample), 65–74 years (12.6%), and ≥785 years (9.1%). Outcomes were lifetime suicidal ideation and attempts. Weighted logistic regression models examined risk and protective factors. Results: Approximately 19% of the sample reported lifetime suicidal ideation, and 6% reported lifetime attempts. Psychological distress, childhood exposure to household mental illness or suicidality, and poor self-rated health were risk factors across all age groups, with associations strengthening with age. Among those with lifetime ideation, these risk factors showed even larger associations in older adults. Protective factors included indicators of sociodemographic vulnerability. Conclusions: Suicide risk and protective factors differ substantially across older adulthood. Findings highlight the need for tailored interventions and integration of suicide screening into primary care for older adults.

## 1. Introduction

In recent years, older adults have experienced significant disparities in suicide rates [1]. People aged 85 or older had the highest suicide rates of any age group in 2023 (22.7 per 100,000 population), and the fastest growth in suicide rates was among older adults (≥65) from 2021 to 2022 [2,3]. Emerging research suggests that suicide risk factors that are most significant among older adults are distinct from those of younger adults. These include depression, poor physical health, functional impairment, pain, cognitive deficits, and social isolation or loneliness [4]. Prior suicide attempts, on the other hand, appear to be not as prominent a risk factor for older adults compared with younger adults [5,6]. At a population level, major suicide prevention efforts tend to target classic risk factors for suicide (e.g., opioid use, firearm ownership) [7]. But these targets may not necessarily address the specific risk factors of older adults, leading to under-identification of suicide risk and inequities in the benefits of public prevention efforts for this population.

In addition to age-related differences, there is also heterogeneity in suicide risk on the basis of race and ethnicity [8]. Trauma, discrimination, psychiatric disorders (particularly depression, anxiety) and sociodemographic factors (lower income, lower educational attainment) are known risk factors for suicidality among racial and ethnic minority adults [8]. Interpersonal factors such as family conflict, intimate partner violence, and perceived burdensomeness are also suicide risk factors that particularly affect racial and ethnic minorities [8,9]. Access to mental healthcare is an important factor in suicide prevention, but racial minorities underutilize mental healthcare compared to their non-Hispanic White counterparts. This is due in part to modifiable barriers to mental healthcare access such as language differences, lack of familiarity with US mental healthcare systems or not knowing how to access them, stigma, shame, embarrassment, and somatization of psychological distress [10].

There is a risk that suicide prevention programs may not be effective or equitable if there is no attention to the specific suicide risk factors that older adults from a diversity of backgrounds experience [7]. Studying protective factors in addition to risk factors for older adult suicides may further illuminate the most impactful intervention targets for this age group. Recent studies have identified unique mental health and suicide protective factors among younger college-age racial minorities, including ethnic identity, cultural individualism, acculturation, and a sense of meaning in life, which are associated with reduced risk [11,12,13]. One study examined character traits that were protective against suicidality among Chinese older adults (e.g., caring, inquisitiveness, self-control) [14], while studies among Black older adults suggest that social support networks and ethnic density are protective factors [8]. However, there is little known about interpersonal or community protective factors that could be targeted with interventions. There is also limited research on suicide protective factors that is specific to older adults from diverse racial/ethnic backgrounds.

Studying risk and protective factors for suicide among older adults could provide directions for future public health and policy efforts to prevent suicide in a group that is currently experiencing suicide disparities. There is also potential that filling this knowledge gap could inform practical strategies to more effectively screen older adults for suicide risk in clinical care. The purpose of this study was to examine risk and protective factors for suicidal ideation and attempts in a racially diverse sample of adults and older adults, stratified by age group (45–64 years, 65–74 years, ≥75 years), from California. Age groups align with typical retirement and pre-retirement ages in the US, as well as age strata used by the Centers for Disease Control and Prevention (CDC) for monitoring suicide trends by age [15].

## 2. Methods

### 2.1. Design and Data

This was a cross-sectional study conducted with (1) data from the California Health Interview Survey (CHIS) from 2021 to 2024, and (2) linked 2020 Centers for Disease Control and Prevention and Agency for Toxic Substances and Disease Registry (CDC/ATSDR) Social Vulnerability Index (SVI) data. CHIS has Institutional Review Board (IRB) approval for investigators from the University of California, Los Angeles to analyze confidential CHIS data through the CHIS Data Access Center. The CHIS Data Access Center performed geocoded data linkage and analyses for enhanced data protection.

The CHIS is a survey of the health and health-related needs of the population of California, conducted continuously since 2001 in two-year cycles [16]. The goal of CHIS is to provide comprehensive data on the health and health needs of the people of California using a population-based sample of more than 20,000 households in the state [16].

The SVI is a geospatial index of four domains (called “themes”) of social vulnerability, derived from 16 US Census variables from the five-year American Community Survey [17]. The four SVI themes are socioeconomic status, household characteristics, racial and ethnic minority status, and housing type/transportation, which combine into a single indicator of place-based social vulnerability. The SVI is constructed at the level of the census tract and has been updated four times from 2000 to 2020 [17]. This analysis used 2020 SVI data, which was most proximal to the CHIS study years, linked to the census tract of CHIS respondents. Additional details about SVI themes are reported below.

### 2.2. Survey Procedures and Sampling Frame

CHIS relies on an address-based sampling frame to survey a representative group of Californians via web or telephone [16]. Households are randomly sampled first, and then one adult in each household is randomly selected to respond to the survey. The sample is designed to include all major racial and ethnic groups in the state, as well as major Asian and Latino ethnic subgroups [18]. The CHIS is offered in English, Spanish, Chinese (Mandarin and Cantonese), Korean, Vietnamese, and Tagalog. It can be completed by phone or web. All CHIS questionnaires and details about its translation protocols are publicly available from the UCLA Center for Health Policy Research [19].

### 2.3. Analytic Sample

Inclusion criteria were adult CHIS participants who completed the survey between 2021 and 2024 and who were 45 years of age or older. The initial pooled sample was 92,397 participants. Fifty-five individuals who were missing data on outcomes were omitted from the analytic sample (0.06% of the sample). Of these, 64,263 were 45 years or older [15].

### 2.4. Measures

Outcomes. The primary outcomes were suicidal ideation (lifetime and past 12 months; “Have you ever seriously thought about committing suicide?” “Have you seriously thought about committing suicide at any time in the past 12 months?”) and past suicide attempts (“Have you ever attempted suicide?”) [20]. These items were asked with a binary yes/no item about ideation first, followed by a binary yes/no item about attempts for those who responded affirmatively to the ideation item.

Exposures. We examined demographic factors, SVI themes, and risk and protective factors for suicidality that were available from CHIS survey measures. Demographic factors were sex, age category (<45, 45–64, 65–74, ≥75), poverty (measured as percentage of the federal poverty line, FPL), health insurance status (insured/not insured), race/ethnicity (Asian, Black, Latino, Native American, Other, White), citizenship status (US citizen, naturalized citizen, non-citizen), household size (one, two, three, four, or five or more members), English language proficiency (only English, speak English very well, speak English not well or not at all), and marital status (married, living with a partner, never married, widowed/separated/divorced).

The four SVI themes, measured at the level of census tracts, were (1) socioeconomic status (poverty, unemployment, housing cost burden, education, uninsurance); (2) household characteristics (older adults, children, disability, single-parent households, English language proficiency); (3) racial and ethnic minority status (Hispanic or Latino, Black or African American, Asian, American Indian or Alaska Native, Native Hawaiian or Pacific Islander, Two or More Races, Other Races); and (4) housing type and transportation (type, crowding, transportation) [17]. Tracts are ranked based on these measures and assigned a percentile value ranging from 0 to 1, with higher values denoting higher levels of social vulnerability.

Suicide risk factors were overall health status (excellent, very good, good, fair, poor), number of chronic illnesses (0–4 from asthma, diabetes, hypertension, and heart disease), past 12-month psychological distress (Kessler Psychological Distress Scale [K6] score ≥ 13) [21], experiencing barriers to mental healthcare (cost of treatment, uncomfortable seeking care, concerned someone would find out, hard to get an appointment), experiencing racial discrimination in healthcare (yes/no), and growing up before age 18 with a household member who had mental illness or suicidality (yes/no), which is considered an adverse childhood experience (ACE) [22]. Suicide protective factors were having stable housing (yes/no) and perceived neighborhood safety (1–4 score based on the average of four Likert-scale neighborhood safety items on the degree to which neighbors are willing to help each other, get along with each other, can be trusted, and overall feeling of safety in the neighborhood).

### 2.5. Analysis

Data were analyzed using R, version 4.4.0 [23]. Weighted frequencies, percentages, and bivariate Chi-square tests were used to describe the sample and compare differences in analytic variables by age group, incorporating CHIS replicate weights to generate population estimates. Age groups of adults and older adults were constructed to align with the age strata the CDC uses for monitoring suicide trends by age [15]. Then, weighted multiple logistic regression models were estimated to examine risk and protective factors for suicide among adults and older adults, stratified by age group. Separate models were estimated for each age group (45–64 years, 65–74 years, ≥75 years) to assess risk for lifetime suicide ideation. Following this, among the subsample who endorsed lifetime suicidal ideation, we examined risk factors for suicide attempts. All models were adjusted for survey year, in addition to including all demographic variables noted above.

## 3. Results

### 3.1. Sample Description

**Figure 1:**
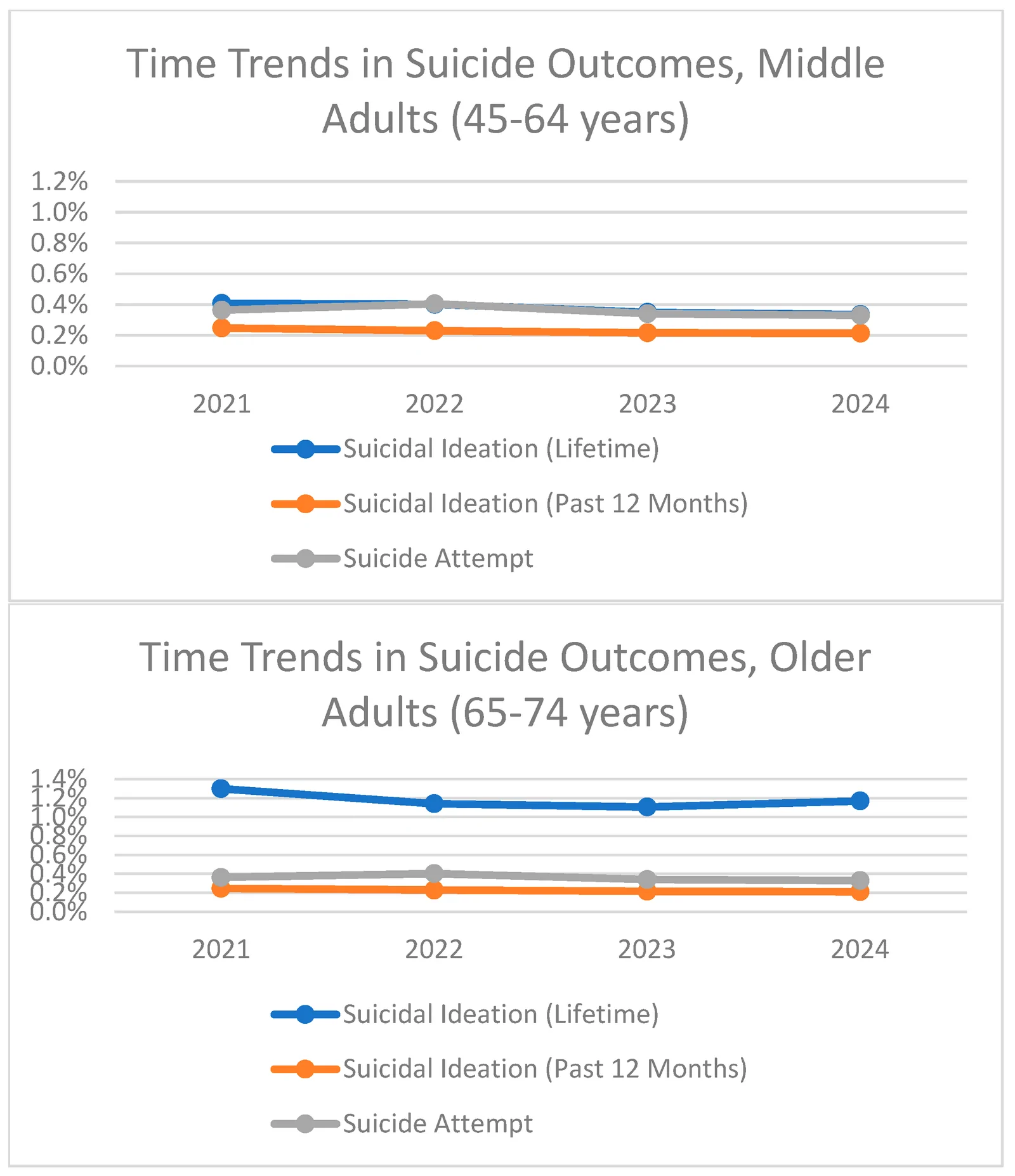

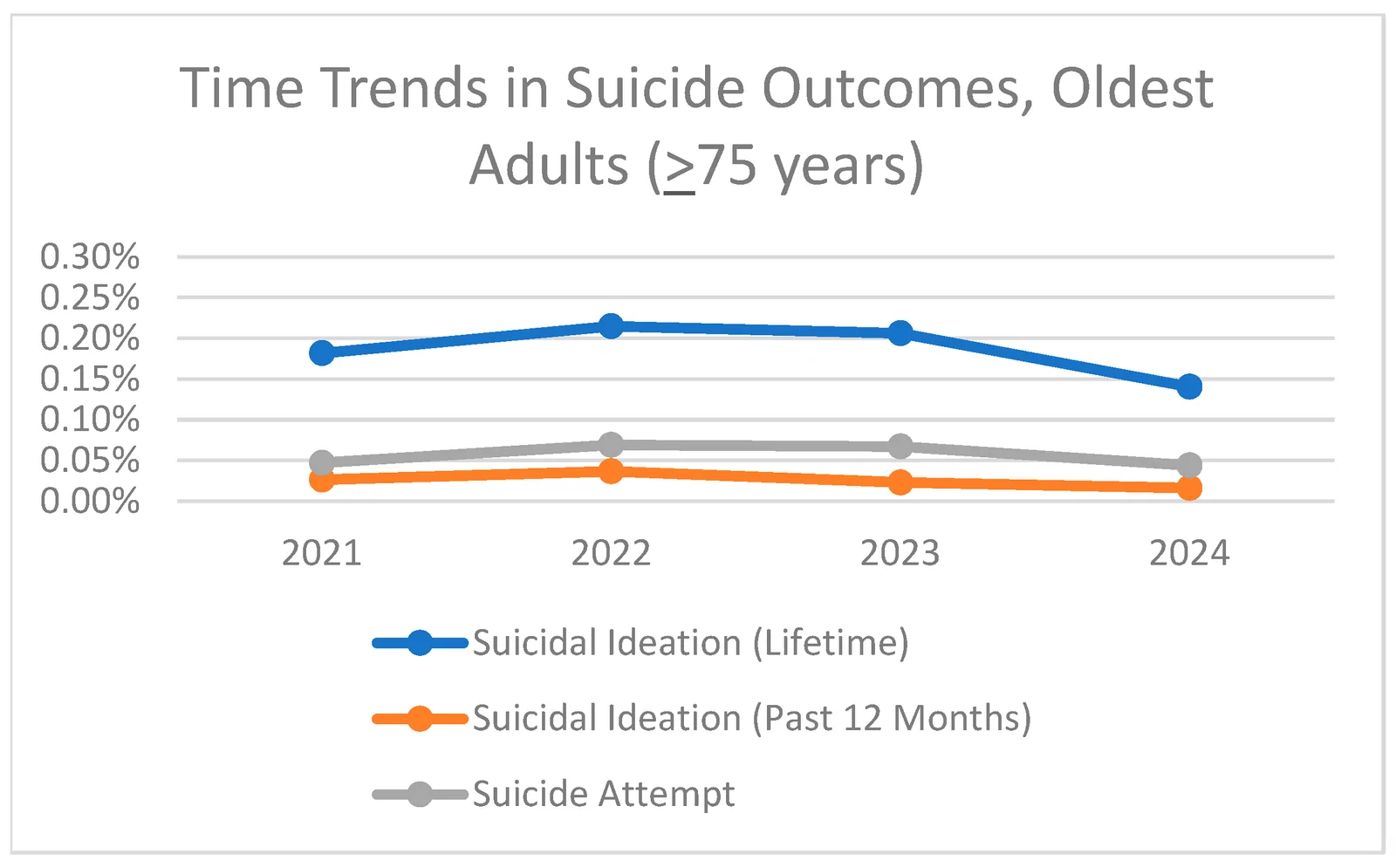
Suicidality over time, CHIS 2021–2024 adult sample. This set of line graphs shows frequency of suicide outcomes over time from 2021 to 2024, stratified by age group, among adults in the 2021–2024 California Health Interview Survey (CHIS). Levels of suicide differed among the three age subgroups. As such, Y-axes are scaled differently for each line graph to allow visualization of within-group trends across time.

The sample was 31.5% middle-aged adults (45–64 years), 19.7% older adults ages 65–84 years, and 2.1% older adults ≥85 years. Older adults in both older-group age categories were more frequently female, living with higher levels of poverty, living alone, and reporting poor or fair overall health, as shown in Table 1. Similarly, all older adults had higher neighborhood safety scores than their younger counterparts and lower overall frequency of suicide outcomes. The overall prevalence of lifetime suicidal ideation was 19.1%, with 5% of the sample reporting past 12-month suicidal ideation and 5.8% reporting a suicidal attempt. Although time trends in the outcomes across the study period (2021–2024) were relatively stable, there was a slight trend towards decreasing prevalence of suicidal ideation (Figure 1). Among older adults ≥85 years, suicidal ideation and attempts rose from 2021 to 2022, and then declined thereafter.

### 3.2. Middle Age Adults (45–64 Years) Risk and Protective Factors

Among adults ages 45–64 years, past-12-month psychological distress and growing up before age 18 with a household member who had suicidality or mental illness were associated with increased risk for lifetime suicidal ideation (aOR = 3.8, 95% CI = 3.31–4.36 and aOR = 2.54, 95% CI = 2.30–2.81, respectively). Additionally, reporting only good, fair, or poor overall health was associated with increased risk (e.g., poor health aOR = 2.06, 95% CI = 1.55–2.73 and fair health aOR = 2.05, 95% CI = 1.70–2.46). Protective factors associated with lower odds of lifetime suicidal ideation were male sex, perceived neighborhood safety score, Latino ethnicity, having a household size of three or four, not being a US citizen, and not speaking English well or at all. SVI theme 3 (racial and ethnic minority status) was also associated with lowered odds of lifetime suicidal ideation. Detailed odds ratios and confidence intervals for these models are shown in Table 2.

Within the subsample of adults in this age group who endorsed lifetime suicidal ideation, as shown in Table 3, growing up before age 18 with a household member who had suicidality or mental illness was associated with a suicide attempt (aOR = 1.39, 95% CI: 1.15–1.67), as was higher social vulnerability in theme 3 (housing composition). Individuals who identified their race/ethnicity as Latino and Other race/ethnicities had higher odds of a suicide attempt when ideation was present. Higher perceived neighborhood safety scores were associated with reduced risk (aOR = 0.80, 95% CI = 0.72–0.89).

### 3.3. Older Adults (65–74 Years) Risk and Protective Factors

There were several significant risk factors for lifetime suicidal ideation among older adults ages 65–74 years (Table 2). These were past-12 month psychological distress (aOR = 3.85, 95% CI = 3.04–4.87), growing up before age 18 with a household member who had suicidality or mental illness (aOR = 3.22, 95% CI = 2.76–3.74), being in only good, fair, or poor overall health (e.g., poor health aOR = 2.19, 95% CI = 1.38–3.46), and cost as a barrier to mental health treatment (aOR = 2.47, 95% CI = 1.41–4.33). The magnitudes of these associations were larger than those of middle-aged adults for the first two variables. Protective factors were male sex, not being a US citizen, and not speaking English well or at all. As observed for middle-aged adults, higher perceived neighborhood safety scores were associated with reduced risk for suicidal ideation (aOR = 0.82, 95% CI = 0.70–0.98).

For the subsample of older adults ages 65–74 who endorsed lifetime suicidal ideation shown in Table 3), there were slight differences in risk and protective factors for suicide attempts compared to middle-aged adults. Being in less than excellent health increased odds of a suicide attempt (e.g., poor health aOR = 3.55, 95% CI = 1.49–8.43), as did concern about being found out as a barrier to mental healthcare (aOR = 4.93, 95% CI = 1.72–14.11). Male sex was associated with reduced odds of a suicide attempt.

### 3.4. Older Adults (≥75 Years) Risk and Protective Factors

Among the oldest adult subsample, past 12-month psychological distress and growing up before age 18 with a household member with mental illness or suicidality were again associated with elevated risk, at larger magnitudes for this age group than the two younger age groups. Having more chronic illnesses was also associated with higher odds of suicidal ideation in this subsample. Finally, cost as a barrier to mental healthcare and discomfort with mental health treatment were associated with 2- and 4-times higher odds of suicidal ideation, respectively, as shown in Table 2. Male sex was associated with reduced odds of suicidal ideation.

For the subsample of older adults who endorsed lifetime suicidal ideation (≥75 years, shown in Table 3), key risk factors associated with higher odds of an attempt were being widowed/divorced/separated, Black race, past 12-month psychological distress, and difficulty getting a mental healthcare appointment. Racial discrimination in healthcare was associated with reduced odds of an attempt in this subsample.

## 4. Discussion

In this study with a large, racially diverse sample of adults in California, we identified risk and protective factors associated with suicidal ideation and attempts, and how they varied across middle and older adulthood. We found that the magnitude of association between several known suicide risk factors (psychological distress, growing up before age 18 with a household member who had mental illness or suicidality, poor self-rated health) became larger within older age strata, even though older adults had lower overall prevalence of suicidality. Another key finding was that several factors typically conceptualized as social disadvantages were inversely associated with suicidal ideation. A recent national study examining county-level suicide and SVI also identified this inverse association between the racial/ethnic minority SVI theme and suicide [24]. Together, these findings suggest that the drivers of suicide risk and factors that confer protection vary across older adulthood. Tailored approaches are needed, including attention to the oldest adults (≥75 years) in this study who had higher levels of risk associated with suicide outcomes (e.g., psychological distress, cost barriers to care).

Neighborhood safety was a consistent protective factor associated with reduced odds of suicidal ideation across age groups [25]. Prior studies have similarly found that neighborhood factors (e.g., crime, traffic, built environment) play a role in the psychological health of older adults, as they directly affect both social and physical health [26]. There were also several demographic factors that were meaningfully associated with reduced suicide risk, including [27,28] Latino ethnicity, non-citizenship, limited English proficiency, and racial/ethnic composition of the community (SVI theme 4). These findings are consistent with hypotheses that cultural norms, cultural beliefs, and familial obligations may buffer against suicide risk [29]. They may reflect what has been described as the “Hispanic paradox,” in which Hispanic and Latino individuals have lower levels of health morbidity than would be expected based on their socioeconomic risk profiles [27,28]. This finding should not be misinterpreted to mean that suicide-related interventions are not a priority for racial and ethnic minority or immigrant older adults; rather, it points to a need for a more granular investigation of within-group risk and protective factors to direct intervention efforts, including neighborhood characteristics. It is also possible that, while these groups have significant psychological distress and life stressors, cultural responses to stress are less likely to include suicidality. Similar to our finding that older adults had lower overall suicidal ideation levels—but among those with suicidal ideation, risk factors for more severe suicide outcomes grew more pronounced with increasing age—there may be subgroups of racial and ethnic minority older adults for whom precision interventions are needed. Male sex was associated with reduced risk of suicide outcomes, pointing to the need to address the mental health needs of older adult women. This pattern is consistent with population patterns of gender differences in suicide, where women report higher rates of ideation and attempts, but men have higher rates of completed suicide [30].

The growing significance of psychological distress and mental health-related adversity in childhood as risk factors with advancing age is consistent with literature documenting that depression, isolation, and accumulated adversity become more salient suicide risk factors in later life [31,32]. Although the prevalence of psychological distress and lifetime suicidal ideation was lower among older adults, risk associations given lifetime ideation were substantially higher than among middle-aged adults. A possible explanation for this finding is that stressful experiences from earlier in life are compounded as new stressors are encountered across the life course, and that there are unique sources of psychological distress affecting older adults. This pattern is concerning given that older adults disclose suicidal intent less frequently than younger adults and there are documented barriers to suicide and mental health screening among older adults in primary care [33,34,35]. The strong association between childhood exposure to a household member with mental illness or suicidality and suicidal ideation later in life also suggests that early life family adversity has cumulative consequences that persist and even intensify later in life, rather than attenuating with time [22,36]. Poor self-rated health was consistently associated with suicide risk across adult and older age groups. This finding aligns with prior work identifying physical health decline, functional impairment, and pain as suicide risk factors specific to later life [37,38]. Because of the significance of physical health as a risk factor for suicidality among older adults, integrating suicide risk assessment and response into primary care is a priority for older adults [30,39,40,41].

Lastly, several barriers to mental healthcare access were associated with suicide risk, including the cost of care (significant across all age strata) and feeling uncomfortable about ideation, and concern about being found out for attempts among older adults. Older adults, who are more likely to live alone and to have smaller social networks, may have concerns about stigma. This, in turn, may reduce their perception that it is worthwhile to seek formal help for mental healthcare [42]. Despite most older adults having insurance via Medicare, cost remains an important barrier to treatment, and this finding points to a need to more closely examine out-of-pocket cost burden among Medicare beneficiaries, as there is evidence for higher cost burden for older adults with mental health needs [43]. Together, these findings highlight the importance of clinical risk assessment among older adults and consideration for how mental healthcare can be delivered in ways that are acceptable, affordable, private, and accessible to older adults.

There were strengths and limitations to this study. The study used a large, population-based sample that allowed for stratification by age categories and detection of age-specific risk and protective factors. We linked geospatial data on social vulnerability within census tracts, which adjusts for potential place-based confounding factors in the social environment. There are also study limitations. The design was cross-sectional, and as a survey, all measures were self-reported. Recall bias could lead to under-estimation of suicide outcomes, and thus estimates may be conservative. The study cannot establish causality, and as an associative study, reverse causality is a possible explanation for the associations we observed. The CHIS sample was community-dwelling Californians and excluded adults in skilled nursing facilities, assisted living, and other institutional settings, which may underrepresent older adults with more significant medical and social risk factors. Suicide risk and protective factors were limited to those available in CHIS, and we were unable to examine a broader set of factors known to be relevant for older adults, such as cognitive status, loneliness, social support, sense of purpose, or religiosity. Residual confounding is also possible related to omitted variables bias, which may reduce the precision of estimates. There were some independent variables in models with the oldest subsample that had wide confidence intervals, and thus estimates should be interpreted with caution for imprecision.

## 5. Conclusions

In a large, racially diverse sample of California adults, suicide risk and protective factors varied substantially across middle and older adulthood. Psychological distress, childhood exposure to household mental illness or suicidality, and poor health as risk factors for suicide became larger with increasing age, while neighborhood safety and several ethnicity-related factors were protective. These findings point to a need to consider how to offer tailored support for mental well-being to older adults who are most at risk and to identify additional protective factors. Findings around mental and physical health as risk factors for suicide among older adults suggest a need for policy efforts to integrate suicide screening and interventions in care of older adults, across both primary care and specialty settings. Future research should examine these patterns of findings longitudinally and test interventions that address the unique drivers of suicide risk among older adults from diverse backgrounds.

## Figures and Tables

**Table 1:** Sample description, CHIS 2021–2024 (*N* = 64,263).

Demographics	Overall 53.3%	Middle (45–64) 31.5%	Older (65–74) 12.6%	Oldest (≥75) 9.1%	*p*-Value
Sex					<0.001
Male	51.2%	30.1%	11.5%	7.7%	
Female	48.8%	33.1%	13.7%	10.7%	
Has health insurance	93.6%	31.5%	13.4%	9.7%	<0.001
Poverty level					<0.001
400% FPL	57.5%	33.6%	13.2%	8.7%	
100% FPL	14.4%	27.2%	9.7%	9.5%	
200% FPL	15.7%	29.1%	12.3%	9.7%	
300% FPL	12.4%	30.2%	13.3%	9.9%	
Race/ethnicity					<0.001
Asian	15.6%	31.7%	10.5%	6.9%	
Black	5.7%	35.1%	14.2%	8.3%	
Latino	23.0%	31.2%	7.5%	3.7%	
Native American	0.8%	33.0%	12.5%	10.7%	
Other	15.1%	31.0%	8.4%	4.9%	
White	39.9%	31.3%	17.7%	14.9%	
Citizenship					<0.001
US-born citizen	67.6%	27.0%	13.0%	9.9%	
Naturalized citizen	19.5%	44.8%	16.3%	11.1%	
Non-citizen	12.9%	35.0%	4.7%	2.5%	
Household size					<0.001
1 member	11.4%	25.6%	21.1%	23.9%	
2 members	31.2%	27.8%	20.1%	14.6%	
3 members	23.1%	34.9%	10.1%	4.8%	
4 members	18.8%	37.6%	4.6%	2.4%	
5 or more members	15.6%	30.9%	4.4%	2.1%	
Marital status					<0.001
Living with a partner	9.1%	23.8%	5.7%	3.0%	
Married	49.6%	42.1%	15.6%	9.0%	
Never married	26.7%	12.1%	3.8%	1.2%	
Widowed, divorced, separated	14.5%	36.1%	22.7%	27.9%	
English language proficiency					<0.001
Not well or none	9.7%	42.8%	15.1%	11.6%	
Speak only English	55.0%	31.3%	15.7%	12.2%	
Speak English very well	35.2%	28.8%	6.9%	3.7%	
Outcomes					
Suicidal ideation (lifetime)	19.1%	24.7%	7.8%	3.9%	<0.001
Suicidal ideation (past 12-month)	5%	18.2%	3.9%	2.0%	<0.001
Suicide attempt	5.8%	24.8%	7.1%	3.9%	<0.001
Suicide Risk/Protective Factors					
Racial discrimination in healthcare	8.1%	31.7%	7.5%	4.1%	<0.001
Past 12-month psychological distress	15.2%	19.1%	4.1%	2.2%	<0.001
Growing up with a household member with mental illness or suicidality	22.1%	27.0%	9.6%	4.3%	<0.001
Overall self-reported health					<0.001
Excellent	17.4%	28.0%	9.5%	4.8%	
Fair	14.4%	35.5%	14.8%	13.6%	
Good	31.3%	32.2%	13.2%	9.7%	
Poor	2.9%	34.5%	16.9%	17.7%	
Very good	34.1%	30.8%	12.3%	8.3%	
Chronic illnesses					<0.001
0	50.8%	28.9%	7.1%	3.6%	
1	32.9%	33.8%	14.9%	11.5%	
2	12.9%	35.6%	23.8%	20.5%	
3	3.1%	34.3%	27.6%	27.2%	
4	0.4%	37.8%	34.1%	20.9%	
Barriers to mental healthcare					
Cost of treatment	5.6%	18.0%	2.7%	1.0%	<0.001
Not comfortable	4.4%	17.9%	3.9%	1.3%	<0.001
Concerned someone will find out	3.2%	17.6%	2.9%	1.5%	<0.001
Hard to get appointment	3.2%	22.4%	3.6%	1.7%	<0.001
Stable housing	95.1%	31.6%	12.8%	9.4%	<0.001
Perceived neighborhood safety score (M, SD, Range: 1–4)	3.05 (0.002)	3.07 (0.004)	3.19 (0.04)	3.26 (0.007)	<0.001

Notes. Weighted percentages comparing differences in demographic factors, suicide outcomes, and suicide risk and protective factors among adults stratified by age group, using pooled data from the 2021–2024 California Health Interview Survey (CHIS). FPL = Federal Poverty Line.

**Table 2:** Correlates of lifetime suicidal ideation among Californian adults, 2021–2024.

	Middle Adults (45–64)			Older Adults (65–74)			Oldest Adults (≥75)		
	aOR	95% CI (L)	95% CI (U)	aOR	95% CI (L)	95% CI (U)	aOR	95% CI (L)	95% CI (U)
Demographics									
Male sex (ref: female)	0.88	0.79	0.98	0.77	0.66	0.90	0.62	0.47	0.81
Race/ethnicity									
Asian (ref: white)	1.00	0.83	1.20	0.75	0.52	1.08	0.51	0.24	1.09
Black	0.84	0.68	1.04	0.72	0.53	0.97	0.98	0.62	1.54
Latino	0.73	0.61	0.87	0.78	0.55	1.11	0.96	0.33	2.80
Native American	1.00	0.49	2.04	1.27	0.68	2.38	1.90	0.98	3.71
Other	0.83	0.71	0.98	0.80	0.62	1.03	1.45	0.91	2.30
Household size									
2 members (ref: 1 member)	1.01	0.88	1.16	0.79	0.63	0.98	0.77	0.55	1.06
3 members	0.76	0.64	0.89	0.96	0.70	1.30	0.66	0.34	1.31
4 members	0.75	0.62	0.89	0.76	0.52	1.10	0.39	0.10	1.56
5 or more members	0.88	0.72	1.08	0.61	0.34	1.11	0.70	0.32	1.54
Poverty									
100% FPL (ref: 400% FPL)	0.81	0.66	0.98	1.00	0.71	1.41	1.14	0.64	2.02
200% FPL	0.90	0.73	1.10	0.89	0.65	1.22	0.95	0.58	1.56
300% FPL	0.96	0.82	1.12	0.98	0.73	1.31	1.08	0.67	1.74
Citizenship status									
Naturalized citizen (ref: citizen)	0.58	0.50	0.68	0.61	0.43	0.87	0.90	0.55	1.48
Non-citizen	0.56	0.44	0.73	0.65	0.36	1.18	0.68	0.29	1.57
English language ability									
Not well/none (ref: only English)	0.72	0.55	0.95	0.49	0.29	0.83	0.40	0.15	1.02
Very well	1.04	0.90	1.20	0.88	0.67	1.15	1.10	0.76	1.60
Marital status									
Living with partner (ref: Married)	1.36	1.15	1.62	1.07	0.75	1.52	1.03	0.40	2.67
Never married	1.27	1.09	1.48	1.25	0.90	1.72	0.98	0.57	1.70
Widowed, separated, divorced	1.30	1.14	1.48	1.18	0.95	1.46	0.90	0.61	1.31
Census Tract SVI (higher = more vulnerable)									
Theme 1 Socioeconomic	1.11	0.85	1.45	1.26	0.84	1.91	1.19	0.60	2.34
Theme 2 Household composition	0.98	0.79	1.21	0.61	0.43	0.84	0.97	0.52	1.81
Theme 3 Minority status/language	0.74	0.58	0.94	0.71	0.48	1.04	0.77	0.46	1.29
Theme 4 Housing/transportation	1.36	1.11	1.66	1.31	0.95	1.80	1.07	0.70	1.63
Suicide Risk/Protective Factors									
Racial discrimination in healthcare	1.19	0.98	1.43	1.29	0.94	1.78	1.29	0.80	2.08
Past 12-month psychological distress	3.80	3.31	4.36	3.85	3.04	4.87	5.89	3.43	10.12
Growing up before age 18 with household member mental illness or suicide	2.54	2.30	2.81	3.22	2.76	3.74	2.97	2.34	3.78
Overall Health Status									
Fair (ref: Excellent)	2.05	1.70	2.46	1.93	1.37	2.73	0.97	0.62	1.51
Good	1.65	1.43	1.91	1.48	1.13	1.95	1.21	0.82	1.78
Poor	2.06	1.55	2.73	2.78	1.81	4.26	1.36	0.72	2.57
Very good	1.30	1.13	1.49	0.96	0.73	1.25	1.05	0.71	1.53
Count of chronic illnesses (0–4)	1.03	0.98	1.09	1.01	0.93	1.10	1.16	1.02	1.32
Barriers to mental healthcare									
Cost of treatment	1.83	1.34	2.49	2.47	1.41	4.33	2.44	1.26	4.74
Not comfortable	1.32	0.93	1.87	1.07	0.54	2.12	4.44	1.99	9.90
Concerned someone will find out	1.48	0.98	2.22	0.90	0.45	1.81	1.23	0.42	3.63
Hard to get appointment	1.18	0.84	1.67	1.04	0.55	1.97	1.34	0.66	2.72
Stable housing	0.91	0.74	1.12	1.02	0.77	1.35	1.02	0.49	2.09
Perceived neighborhood safety score (1–4)	0.80	0.72	0.89	0.82	0.70	0.98	0.84	0.63	1.12

Notes. This table shows weighted multiple logistic regression models examining correlates of suicidal ideation among three age groups of adults and older adults (45–64, 65–74, ≥75). Models were estimated separately for each age group. The sample was adult participants in the California Health Interview Survey (CHIS) pooled from 2021 to 2024. FPL = Federal Poverty Line. All estimates incorporated CHIS replicate weights and were adjusted for study year and health insurance status.

**Table 3:** Correlates of lifetime suicide attempts among Californian adults with suicidal ideation, 2021–2024.

	Middle Adults (45–64)			Older Adults (65–74)			Oldest Adults (≥75)		
	aOR	95% CI (L)	95% CI (U)	aOR	95% CI (L)	95% CI (U)	aOR	95% CI (L)	95% CI (U)
Demographics									
Male sex (ref: female)	0.84	0.69	1.01	0.61	0.44	0.85	0.87	0.45	1.68
Race/ethnicity									
Asian (ref: white)	1.02	0.71	1.48	0.80	0.39	1.65	1.88	0.44	8.11
Black	1.26	0.84	1.87	0.61	0.25	1.49	4.78	1.77	12.89
Latino	1.40	1.01	1.95	0.89	0.50	1.57	0.50	0.06	4.46
Native American	0.85	0.34	2.13	0.36	0.13	1.01	0.73	0.03	18.15
Other	1.47	1.13	1.91	0.94	0.58	1.51	0.87	0.27	2.76
Household size									
2 members (ref: 1 member)	0.99	0.77	1.26	1.00	0.66	1.51	2.24	0.95	5.30
3 members	0.89	0.66	1.21	0.74	0.42	1.28	2.06	0.64	6.68
4 members	0.98	0.67	1.44	1.00	0.50	2.00	11.07	1.03	118.55
5 or more members	0.75	0.50	1.12	2.50	0.87	7.16	1.77	0.11	28.92
Poverty									
100% FPL (ref: 400% FPL)	0.89	0.63	1.25	1.39	0.81	2.38	0.46	0.17	1.22
200% FPL	0.68	0.46	1.00	0.69	0.36	1.33	0.41	0.14	1.21
300% FPL	0.53	0.39	0.73	1.03	0.58	1.84	0.37	0.15	0.90
Citizenship status									
Naturalized citizen (ref: citizen)	1.21	0.88	1.68	1.24	0.77	2.01	1.40	0.42	4.69
Non-citizen	1.29	0.79	2.10	0.32	0.07	1.42	0.94	0.18	4.82
English language ability									
Not well/none (ref: only English)	0.69	0.40	1.19	1.71	0.63	4.66	2.00	0.28	14.32
Very well	0.93	0.72	1.22	1.13	0.69	1.85	0.64	0.23	1.74
Marital status									
Living with partner (ref: Married)	1.58	1.13	2.21	0.94	0.46	1.92	1.53	0.21	11.19
Never married	1.25	0.90	1.75	0.92	0.52	1.63	3.28	0.90	11.96
Widowed, separated, divorced	1.11	0.86	1.42	1.25	0.79	1.98	2.69	1.10	6.58
Census Tract SVI (higher = more vulnerable)									
Theme 1 Socioeconomic	1.28	0.77	2.13	1.51	0.55	4.14	1.32	0.32	5.45
Theme 2 Household composition	1.85	1.22	2.81	1.09	0.55	2.17	2.69	0.66	10.97
Theme 3 Minority status/language	0.93	0.61	1.41	0.99	0.44	2.26	1.49	0.48	4.62
Theme 4 Housing/transportation	1.03	0.69	1.53	1.37	0.73	2.56	0.27	0.09	0.76
Suicide Risk/Protective Factors									
Racial discrimination in healthcare	0.87	0.66	1.17	1.14	0.62	2.09	0.15	0.05	0.46
Past 12-month psychological distress	1.08	0.88	1.33	1.29	0.84	1.96	2.76	1.18	6.46
Growing up before age 18 with household member mental illness or suicide	1.39	1.15	1.67	1.15	0.83	1.59	0.86	0.45	1.66
Overall Health Status									
Fair (ref: Excellent)	1.20	0.87	1.67	1.93	0.94	4.00	0.70	0.24	2.06
Good	1.00	0.76	1.33	2.19	1.13	4.22	0.48	0.18	1.28
Poor	1.15	0.70	1.91	3.55	1.49	8.43	1.64	0.41	6.49
Very good	0.77	0.57	1.03	1.61	0.85	3.04	0.46	0.16	1.29
Count of chronic illnesses (0–4)	1.02	0.92	1.14	1.06	0.92	1.22	1.29	0.96	1.72
Barriers to mental healthcare									
Cost of treatment	0.87	0.57	1.32	1.05	0.47	2.34	0.35	0.06	2.17
Not comfortable	0.81	0.50	1.31	0.20	0.08	0.52	0.62	0.13	3.04
Concerned someone will find out	0.53	0.31	0.93	4.93	1.72	14.11	1.50	0.16	13.73
Hard to get appointment	0.71	0.48	1.05	1.62	0.67	3.93	6.88	1.16	40.94
Stable housing	1.02	0.74	1.40	0.71	0.40	1.26	0.47	0.05	4.33
Perceived neighborhood safety score (1–4)	0.80	0.68	0.94	0.88	0.63	1.22	1.53	0.78	3.00

Notes. This table shows weighted multiple logistic regression models examining correlates of suicide attempts among three age groups of adults and older adults (45–64, 65–74, ≥75). Models were estimated separately for each age group and each outcome. The sample was adult participants in the California Health Interview Survey (CHIS) pooled from 2021 to 2024. FPL = Federal Poverty Line. All estimates incorporated CHIS replicate weights and were adjusted for study year and health insurance status.

## Data Availability

CHIS data are available through the UCLA Center for Health Policy Research (https://healthpolicy.ucla.edu/our-work/california-health-interview-survey-chis, accessed on 6 August 2026).
